# Patient-Reported Outcome Measures and Clinical Outcomes in Children with Foregut Anomalies

**DOI:** 10.3390/children8070587

**Published:** 2021-07-10

**Authors:** Isabel I. Sreeram, Chantal A. ten Kate, Joost van Rosmalen, Johannes M. Schnater, Saskia J. Gischler, René M. H. Wijnen, Hanneke IJsselstijn, André B. Rietman

**Affiliations:** 1 Department of Pediatric Surgery and Intensive Care, Erasmus MC Sophia Children’s Hospital, P.O. Box 2060, 3000 CB Rotterdam, The Netherlands; i.sreeram@erasmusmc.nl (I.I.S.); c.tenkate@erasmusmc.nl (C.A.t.K.); j.schnater@erasmusmc.nl (J.M.S.); s.gischler@erasmusmc.nl (S.J.G.); r.wijnen@erasmusmc.nl (R.M.H.W.); h.ijsselstijn@erasmusmc.nl (H.I.); 2 Department of Biostatistics, Erasmus University Medical Centre, P.O. Box 2060, 3000 CB Rotterdam, The Netherlands; j.vanrosmalen@erasmusmc.nl; 3 Department of Epidemiology, Erasmus University Medical Centre, P.O. Box 2060, 3000 CB Rotterdam, The Netherlands; 4 Department of Child and Adolescent Psychiatry/Psychology, Erasmus MC Sophia Children’s Hospital, P.O. Box 2060, 3000 CB Rotterdam, The Netherlands

**Keywords:** congenital diaphragmatic hernia, esophageal atresia, congenital lung malformations, value-based healthcare, clinical decision-making

## Abstract

Increasing numbers of children and adults with chronic disease status highlight the need for a value-based healthcare system. Patient-reported outcome measures (PROMs) are essential to value-based healthcare, yet it remains unclear how they relate to clinical outcomes such as health and daily functioning. We aimed to assess the added value of self-reported PROMs for health status (HS) and quality of life (QoL) in the long-term follow-up of children with foregut anomalies. We evaluated data of PROMs for HS and/or QoL among eight-year-olds born with congenital diaphragmatic hernia (CDH), esophageal atresia (EA), or congenital lung malformations (CLM), collected within the infrastructure of a multidisciplinary, longitudinal follow-up program. Clinical outcomes were categorized into different outcome domains, and their relationships with self-reported HS and QoL were assessed through multivariable linear regression analyses. A total of 220 children completed HS and/or QoL self-reports. In children with CDH and EA, lower cognition was significantly associated with lower self-reported HS. Due to the low number of cases, multivariable linear regression analysis was not possible in children with CLM. HS, QoL, and clinical outcomes represent different aspects of a child’s wellbeing and should be measured simultaneously to facilitate a more holistic approach to clinical decision making.

## 1. Introduction

Survival among children with congenital anomalies has greatly improved in recent decades. As a result, the numbers of children and adults living with a chronic condition are steadily increasing, causing significant financial strain on healthcare systems [1,2,3]. To optimize economic sustainability while improving health, emphasis must be placed on value-based healthcare (VBHC) [4]. This can be achieved by focusing on the impact of disease-related morbidities on a patient’s self-perceived health status (HS) and quality of life (QoL) [4,5,6]. Patient-reported outcome measures (PROMs) are widely used tools for assessing long-term outcomes and are essential to value-based decision making [5,7]. However, it remains unclear how PROMs relate to clinical outcomes such as health, morbidity, and daily functioning in children with chronic conditions [5].

Our tertiary level European center of expertise for children born with congenital anomalies offers all children born with congenital anatomical anomalies a standardized multidisciplinary long-term follow-up program [8]. This unique infrastructure allows us to measure children’s perceptions of their HS and QoL in a standardized manner, and to simultaneously monitor clinical outcomes throughout childhood [9,10].

To date, associations between clinical outcomes and self-perceived HS and QoL have never been investigated in children born with anatomical anomalies. Recognition of any relationship could serve to aid clinical decision making with respect to a given child’s own experiences. In time, this might help to optimize the value of healthcare from the perspectives of cost-effectiveness and patient compliance.

We hypothesized that impairments in clinical outcomes would be associated with lower self-reported HS, but not with lower QoL. In this study, we aimed to assess the added value of PROMs for HS and QoL in the long-term follow-up of eight-year-old children with congenital foregut anomalies. Therefore, we studied the relationship between clinical outcomes and self-reported HS and QoL by evaluating clinical data from our longitudinal follow-up program.

## 2. Materials and Methods

### 2.1. Study Population

We analyzed available data from children born with congenital diaphragmatic hernia (CDH), esophageal atresia (EA), or congenital lung malformations (CLM) between January 1999 and December 2012, who joined our standardized prospective longitudinal follow-up program for children with congenital anomalies [8] and who underwent surgery within the first 28 days of life. Children with CLM were only included if they had undergone surgical resection of the lesion ≤28 days after birth. Data were collected until June 2020. The Medical Research Involving Human Subjects Act did not apply to this study, as stated by the Institutional Ethics Review Board (MEC-2020-0551).

### 2.2. Data Collection

For this study, we selected children who completed a Pediatric Quality of Life Inventory (PedsQL, a PROM for HS) and/or Dutch-Child-AZL-TNO-Quality-of-Life (DUX-25, a PROM for QoL) questionnaire during follow-up assessments at eight years of age. Standardized assessments of health and daily functioning were performed as standard of care using previously described instruments and methods [11,12,13,14]. Although our follow-up protocol was subject to changes in regular care throughout the study period, evaluation methods were considered interchangeable and the same outcomes were measured over the years.

Clinical outcomes were categorized into 14 domains (cognition, behavior, daily executive functioning, motor function, maximum exercise capacity, lung function, presence of gastroesophageal reflux (GER), respiratory morbidity, daily use of medication for either a physical or psychological condition, presence of scoliosis, need for tube feeding, and need for home oxygen), modified from previous outcome classification tables [10,15]. Apart from behavior and daily executive functioning, all domains were assessed directly by our multidisciplinary follow-up team. A detailed description of all clinical outcome domains, instruments, and cut-off values is provided in the Supplementary Materials (Supplementary Materials S1).

During neuropsychological assessments, children filled in PedsQL and DUX-25 self-reports. Cognition was measured through intelligence testing. Emotional and behavioral problems (summarized as ‘behavior’) and daily executive functioning were evaluated using parent-proxy reports. Motor function was measured with the movement assessment battery for children. Maximum exercise capacity was evaluated using a treadmill test according to the Bruce protocol.

Lung function was tested using spirometry. Presence of GER was routinely determined by 24 h pH-impedance testing in children with CDH and EA. Respiratory morbidity was defined as ≥3 lower respiratory tract infections in the past year requiring antibiotic therapy and/or hospitalization, or presence of vocal cord dysfunction or subglottic stenosis requiring regular follow-up. Treatment modalities affecting daily life, such as daily use of medication for physical and/or psychological conditions and the need for tube feeding and/or home oxygen, were registered. Scoliosis was defined as present when requiring regular follow-up and/or surgical intervention.

The following background data were retrieved from electronic patient records: sex, gestational age, birth weight, type and specification of anomaly [16,17], presence of associated problems, type of primary surgery, duration of anesthetic exposure within the first 24 months of life, educational level, and highest maternal educational level (MEL). Preterm birth was defined as gestational age <37 weeks. Small for gestational age was defined as birth weight <10th percentile [18]. Duration of anesthetic exposure was defined as the time between induction and departure from the operating theater. VACTERL (vertebral, anorectal, cardiac, tracheoesophageal, renal, and limb malformations) association in children with EA was defined according to Solomon [19]. Associated anomalies were considered major if surgical intervention or regular hospital visits were required. MEL was recorded as a proxy for socioeconomic status.

### 2.3. Data Analysis

Data are presented as frequencies (%) or median (interquartile range). Background data were compared between participants and non-participants using Mann–Whitney-U tests or Fisher’s exact tests. Clinical outcomes were categorized as normal, borderline, or impaired (cognition, behavior, daily executive functioning), as normal or impaired (motor function, maximum exercise capacity, lung function), or as yes or no (GER, respiratory morbidity, daily use of medication for physical/psychological condition, scoliosis, tube feeding, home oxygen). See Supplementary Materials for appropriate cut-off values (Supplementary Materials S1).

To assess the relationship between the different domains and the PedsQL and DUX-25 scores, multivariable linear regression analyses were performed for each diagnostic group. All PedsQL and DUX-25 subscales were selected as dependent variables, whereas cognition, behavior, daily executive functioning, motor function, maximum exercise capacity, lung function, and GER were selected as candidate independent variables. Subsequently, multiple imputation using fully conditional specification was implemented for all candidate independent variables, using 30 iterations and 50 imputations. Results were pooled over imputed data sets according to Rubin’s rules. Variables with >35% missing values were excluded from the regression analysis. As a result, the regression models included the following independent variables: cognition, behavior, motor function, maximum exercise capacity, and lung function. For the final analyses, sex and MEL were added to the independent variables in order to rule out potential bias. Results are summarized as regression coefficients (B), 95% confidence intervals (CI), and *p*-values. A *p*-value < 0.05 was considered to indicate significance. All data were analyzed using SPSS version 25.0 (IBM, Chicago, IL, USA).

## 3. Results

### 3.1. Patient Characteristics

**Figure 1:**
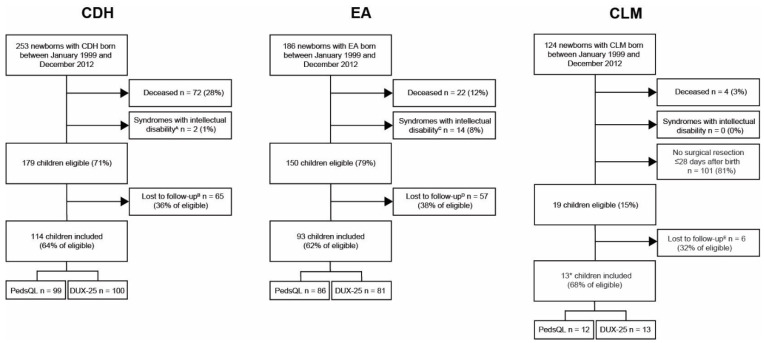
Flowcharts of included patients. CDH = congenital diaphragmatic hernia, EA = esophageal atresia, CLM = congenital lung malformation. ^A^ Wolf-Hirschhorn syndrome n = 1, Simpson–Golabi–Behmel syndrome n = 1. ^B^ emigration n = 5, organizational reasons n = 27, no neuropsychological assessment n = 7, no follow-up scheduled at 8 years n = 2, no self-reports due to lack of time n = 2, age ≥9 years due to postponement of follow-up visit n = 1, refusal n = 18, follow-up elsewhere n = 1, untraceable n = 2. ^C^ Down syndrome n = 5, Opitz syndrome n = 1, Goldenbar syndrome n = 1, Wolf–Hirschhorn syndrome n = 1, Mandibulofacial dysostosis Guion Almeida type n = 1, 22q11 duplication syndrome n = 1, other n = 4. ^D^ emigration n = 6, organizational reasons n = 4, no neuropsychological assessment n = 6, no follow-up scheduled at 8 years n = 8, no self-reports due to lack of time n = 8, age ≥9 years due to postponement of follow-up visit n = 4, refusal n = 12, follow-up elsewhere n = 5, untraceable n = 4. ^E^ organizational reasons n = 2, no self-reports due to lack of time n = 1, refusal n = 2, untraceable n = 1. * One child with CDH had an incidental finding of CLM and was not included in the CLM group.

We identified 563 children with CDH, EA, or CLM born between 1999 and 2012, of whom 83% had survived. Of the survivors, 15 children (3%) had syndromes with severe intellectual disability, 101 children with CLM had no surgery within 28 days after birth, and 1 child with CDH had an incidental finding of CLM, and was only analyzed in the CDH group, leaving 350 eligible children. A total of 220 children (63% of eligible number) completed the PedsQL and/or DUX-25 at 8 years of age (CDH n = 114, EA n = 93, CLM n = 13) (Figure 1). Participants had a median age of 8.2 years (range 7–9), and clinical characteristics did not differ significantly between participants and non-participants across all three diagnostic groups (Table 1). In line with official data from the Statistics Netherlands’ database, [20] 209 children (95%) attended regular education.

### 3.2. CDH

The majority of the 114 children with CDH scored normal on cognition, behavior, daily executive functioning, motor function, and lung function. Out of these these 114 children, 98 (86%) scored below normal scores for at least 1 of the 14 clinical outcome domains. Normal maximum exercise capacity was observed in 42% of children. GER was absent in the majority of children who underwent routine 24 h pH-impedance testing. See Table 2 for more details on clinical outcomes.

**Figure 2:**
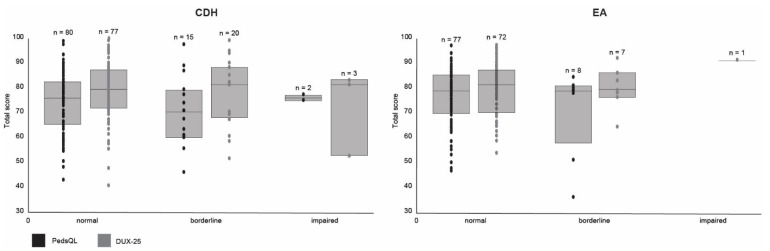
PedsQL and DUX-25 scores for normal, borderline, and impaired cognition. CDH = congenital diaphragmatic hernia, EA = esophageal atresia. Boxes indicate the interquartile range with median.

Lower cognition was significantly associated with lower self-reported HS (Table 3). Figure 2 presents the raw total PedsQL and DUX-25 scores for normal, borderline, and impaired cognition. Lower scores on behavior, motor function, maximum exercise capacity, and lung function were not associated with lower self-reported HS. We found no significant associations between clinical outcomes and QoL. These findings persisted after correction for sex and MEL (see Supplementary Materials S2).

### 3.3. EA

The majority of the 93 children with EA scored normal on cognition, behavior, motor function, and lung function. Out of these 93 children, 83 (89%) had below normal scores for at least 1 of the 14 clinical outcome domains. Daily executive functioning was normal in 48% of children, and 46% had a normal exercise capacity. GER was absent in the majority of children who underwent routine 24 h pH-impedance testing. See Table 2 for more details on clinical outcomes.

Lower cognition and behavior were both significantly associated with lower self-reported HS (Table 3). We found no significant associations between any clinical outcome and QoL. After correction for sex and MEL, the significant association between cognition and self-reported HS disappeared (Supplementary Materials S2).

### 3.4. CLM

Out of the 13 children with CLM, 8 (62%) had below normal scores for at least one of the 14 clinical outcome domains. Due to the low number of cases, multivariable linear regression was not possible; therefore, the association between the health domains and self-perceived HS and QoL could not be evaluated in these children.

## 4. Discussion

In this cohort of eight-year-old children born with foregut anomalies, we evaluated the relationship between generic PROMs and clinical outcomes. The standardized assessments of health and daily functioning indicated favorable clinical outcomes overall. In total, 189 (86%) out of 220 children with CDH, EA, or CLM had below normal scores for at least 1 of the 14 clinical outcome domains. In children with CDH, only lower cognition was associated with lower patient-reported HS. In children with EA, lower cognition and behavior were both associated with lower HS; although, after correction for sex and MEL, only the association with behavior remained. We found no associations between clinical outcomes and patient-reported QoL. Our results suggest that disease-related morbidity is not associated with self-reported HS, nor with self-reported QoL in school-aged children.

Determining what matters most to patients is essential to VBHC, the focus of which is improving quality of care while promoting cost-effectiveness [4]. When evaluating disease burden, both the physical and psychosocial impact of disease should be taken into account. Although PROMs play a central role in capturing patient experience, studies on the relationship between clinical outcomes and patient-reported HS and QoL remain scarce, and have mostly focused on clinical characteristics and symptoms rather than long-term outcomes [22,23].

Our findings are similar to those previously reported by our research group for other critical conditions. A study in five-year-old neonatal extracorporeal membrane oxygenation (ECMO) survivors also found a relationship between cognition and HS, although proxy-reports were used instead of self-reports [10]. Another study in eight-year-old neonatal ECMO survivors found no correlation between self-reported HS and actual motor performance, and self-perceived motor performance was found to be better than actual motor performance [9]. By contrast, a study in children with a history of laryngotracheal stenosis found that exercise capacity and lung function were positively associated with HS. However, this relationship was determined using parent-reported questionnaires, the results of which were found to differ significantly from self-reports [24].

In the present study, we found an association between clinical outcome and patient-reported HS, but only for children with CDH and borderline cognition. The lack of relationships between other clinical outcomes and PROMs might be explained by several factors. First, it has been suggested that objective outcome measurements often focus on one physiological aspect, whereas PROMs are multifactorial [25]. Second, school-aged children with congenital anomalies tend to overestimate their performance, a phenomenon known as superiority bias [26]. However, despite the disparity between actual competence and self-reported competence, eight-year-olds have been found to be able to provide valid and reliable self-reports [27].

The general disagreement between clinical outcomes and a child’s own experiences found in our study indicates that identification of long-term morbidities cannot be based solely on PROMs for HS and QoL. Given that clinical outcomes tend to deteriorate with age in children born with foregut anomalies [14,28,29], the importance of routinely measuring clinical outcomes becomes more apparent, as it allows for early identification of children at risk of declining performance. This should be communicated clearly to both children and their caregivers in order to support a discussion about the added value of complying to a timely intervention when problems arise. As such, PROMs may be used to support clinical decision making by prioritizing comorbidities in relation to what matters most to a given child. Clinicians with sufficient knowledge of long-term morbidities and their potential consequences later in life should counsel the child and their family about associated morbidities that do not directly affect self-perceived HS or QoL at school age. Moreover, since the wellbeing of children with lower IQ is particularly influenced by their cognitive disadvantage, clinicians must ensure that a child’s level of academic performance matches their developmental level.

Here, we discuss the association between generic PROMs and clinical outcomes. Since condition-specific PROMs have been shown to provide more sensitive information than generic instruments [30], studies using disease-specific PROMs might provide new insights into this relationship and help define a disease-specific minimal set of outcome measures.

Strengths of our study include the use of a relatively large and representative cohort of children with a rare congenital anomaly, whose data were collected within the infrastructure of a standardized longitudinal follow-up program. Several limitations need to be addressed. First, due to the small number of children with CLM who underwent surgery in the neonatal period, it was not feasible to assess the relationship between PROMs and clinical outcomes for these children. Nevertheless, we decided to show their data in order to cover the outcome results of a homogeneous group of children born with anatomical foregut anomalies. Second, a relatively large amount of data was missing for several clinical outcomes, notably behavior, daily executive functioning, and GER. These outcomes were added to our follow-up program at a later stage and might have caused confounding bias. Finally, our results may be subject to superiority bias. This tendency potentially disappears as children grow older. Longitudinal studies evaluating PROMs at 8 and 12 years of age are currently being performed by our department.

In conclusion, apart from an association between lower cognition or behavioral problems and lower patient-reported HS, we found limited relationships between clinical outcomes and PROMs for HS and QoL in school-aged children with foregut anomalies. Based on these findings, HS, QoL and clinical outcomes should be regarded as different concepts that should be routinely measured in order to facilitate a more holistic approach to clinical decision making. PROMs may be useful to prioritize assessment and treatment of comorbidities in relation to a child’s experiences and, as such, can promote VBHC in children with chronic conditions.

Our study provides a framework to assess the added value of PROMs in clinical decision making. Future studies should aim to broaden this perspective by including other chronic conditions and disease-specific PROMs. A clearer understanding of these relationships will help optimize healthcare systems while improving clinical outcomes, HS, and QoL in children with chronic disease status.

## Figures and Tables

**Table 1:** Demographic variables of participating and non-participating children with congenital diaphragmatic hernia (CDH), esophageal atresia (EA), and congenital lung malformation (CLM). Data are presented as n (%) or median (IQR). One patient had both CDH and an incidental finding of CLM, and was included in the CDH group. CPAM = congenital pulmonary airway malformation, BPS = bronchopulmonary sequestration, CLE = congenital lobar emphysema, BC = bronchogenic cyst, VACTERL = vertebral, anorectal, cardiac, tracheoesophageal, renal, and limb malformations, ISCED = International Classification of Education [21]. Asterisk indicates significance (*p* < 0.05).

	CDH			EA			CLM		
	Participants (n = 114 *)	Non-Participants (n = 65)	*p*-Value	Participants (n = 93)	Non-Participants (n = 58)	*p*-Value	Participants (n = 13)	Non-Participants (n = 6)	*p*-Value
**Baseline characteristics**									
Male	70 (61.4)	36 (55.4)	0.53	57 (61.3)	37 (63.8)	0.45	10 (76.9)	3 (50.0)	0.32
Gestational age in weeks	38.7 (37.7–39.6)	38.6 (38.0–39.9)	0.87	37.9 (36.2–40.0)	38.4 (36.1–40.0)	0.81	38.7 (36.8–40.4)	38.4 (37.5–39.9)	0.58
Birth weight in grams	3000 (2800–3445)	3000 (2503–3480)	0.71	2850 (2160–3180)	2735 (2108–3128)	0.38	3265 (3070–3690)	3088 (2846–3888)	0.42
Preterm birth	14 (12.3)	12 (18.5)	0.18	32 (34.4)	20 (34.5)	0.50	3 (23.1)	1 (16.7)	1.00
Small for gestational age ^A^	28 (24.6)	10 (15.4)	0.55	36 (38.7)	21 (36.2)	0.57	0	1 (16.7)	
Side of hernia									
Left	98 (86.0)	55 (84.6)	0.83						
Right	16 (14.0)	10 (15.4)	0.83						
Type repair									
Primary repair	40 (35.1)	24 (36.9)	0.70						
Patch	73 (64.0)	41 (63.1)	0.70						
Unknown	1 (0.9)	0							
Type of EA ^B^									
Type A				7 (7.5)	6 (10.3)	0.56			
Type B				0	2 (3.4)				
Type C				82 (88.2)	43 (74.1)	0.06			
Type D				0	1 (1.7)				
Type E				3 (3.2)	4 (6.9)	0.43			
Unknown				1 (1.1)	2 (3.4)				
Staged repair				10 (10.8)	12 (20.7)	0.10			
Type of CLM									
CPAM							7 (53.8)	5 (83.3)	0.04
BPS							2 (15.4)	1 (16.7)	1.00
CLE							3 (23.1)	0	
BC							0	0	
Hybrid ^C^							1 (7.7)	0	
Associated problems									
VACTERL ^D^				11 (11.8)	12 (20.7)	0.17			
Major anomalies	26 (22.8)	13 (20.0)	0.71	35 (37.6)	29 (50.0)	0.18	1 (7.7)	0	
Minor anomalies	5 (4.4)	6 (9.2)	0.21	28 (30.1)	23 (39.7)	0.29	0	1 (16.7)	
Type of primary surgery									
Thoracotomy	1 (0.9)	0		63 (67.7)	43 (74.1)	0.36	10 (76.9)	3 (50.0)	1.00
Thoracoscopy	33 (28.9)	22 (33.8)	0.40	28 (30.1)	14 (24.1)	0.58	2 (15.4)	1 (16.7)	1.00
Laparotomy	67 (58.8)	30 (46.2)	0.21	0	0		0	0	
Laparoscopy	1 (0.9)	3 (4.6)	0.13	0	0		0	0	
Converted	10 (8.8)	6 (9.2)	1.00	2 (2.2)	0		0	0	
Unknown ^E^	1 (0.9)	4 (6.2)		0	1 (1.7)		1 (7.7)	2 (33.3)	
Duration of anaesthetic exposure in the first 24 months of life in minutes	270 (184–422)	265 (170–505)	0.90	393 (261–786)	442 (299–798)	0.25	203 (184–229)	210 (58–264)	0.80
** Characteristics at time of FU **									
Age at FU	8.2 (8.1–8.3)			8.2 (8.1–8.3)			8.2 (8.1–8.3)		
Educational level									
Regular	97 (85.1)	26 (40.0)	0.05	71 (76.3)	30 (51.7)	0.53	11 (84.6)	1 (16.7)	0.37
Regular with help	11 (9.6)	4 (6.2)	0.75	17 (18.3)	3 (5.2)	0.12	2 (15.4)	1 (16.7)	0.37
Special education	6 (5.3)	7 (10.8)	0.19	5 (5.4)	7 (12.1)	0.05	0	0	
Other	0	0		0	2 (3.4)		0	0	
Unknown	0	28 (43.1)		0	16 (27.6)		0	4 (66.7)	
Maternal educational level									
Low (ISCED 0–2)	8 (7.0)	2 (3.1)	1.00	15 (16.1)	8 (13.8)	1.00	2 (15.4)	0	
Middle (ISCED 3–4)	43 (37.7)	11 (16.9)	0.53	37 (39.8)	20 (34.5)	0.86	2 (15.4)	1 (16.7)	1.00
High (ISCED 5–8)	47 (41.2)	17 (26.2)	0.53	39 (41.9)	19 (32.8)	0.86	7 (53.8)	3 (50.0)	1.00
Unknown	16 (14.0)	35 (53.8)		2 (2.2)	11 (19.0)		2 (15.4)	2 (33.3)	

^A^ Birth weight <10th percentile [18] .^B^ According to Gross classification [16]. ^C^ Combination of CPAM and BPS. ^D^ According to Solomon criteria [19]. ^E^ Surgery abroad.

**Table 2:** Clinical outcomes of 8-year-old children with congenital diaphragmatic hernia (CDH, n = 114), esophageal atresia (EA, n = 93), and congenital lung malformation (CLM, n = 13), summarized per clinical domain. Data are presented as n (% of total cohort). Cognition, proxy-reported behavior, and proxy-reported daily executive functioning were categorized as normal, borderline, or impaired. All other clinical domains were categorized as either normal or impaired, or as yes or no. See the Methods section and the Supplementary Materials for a detailed description of the administered instruments.

		CDH				EA				CLM			
		Normal	Borderline	Impaired	Missing	Normal	Borderline	Impaired	Missing	Normal	Borderline	Impaired	Missing
**Health and daily functioning**	Cognition	87 (76.3)	21 (18.4)	4 (3.5)	2 (1.8)	82 (88.2)	9 (9.7)	1 (1.1)	1 (1.1)	13 (100.0)	0	0	0
	Proxy-reported behavior ^A^	68 (59.6)	11 (9.6)	15 (13.2)	20 (17.5)	51 (54.8)	15 (16.1)	5 (5.4)	22 (23.7)	8 (61.5)	0	0	5 (38.5)
	Proxy-reported daily ^A^ executive functioning	64 (56.1)	5 (4.4)	2 (1.8)	43 (37.7)	45 (48.4)	2 (2.2)	1 (1.1)	45 (48.4)	7 (53.8)	0	0	6 (46.2)
	Motor function	75 (65.8)		37 (32.5)	2 (1.8)	65 (69.9)		25 (26.9)	3 (3.2)	11 (84.6)		2 (15.4)	0
	Maximum exercise capacity	48 (42.1)		52 (45.6)	14 (12.3)	43 (46.2)		43 (46.2)	7 (7.5)	8 (61.5)		4 (30.8)	1 (7.7)
	Lung function	69 (60.5)		42 (36.8)	3 (2.6)	55 (59.1)		37 (39.8)	1 (1.1)	7 (53.8)		6 (46.2)	0
		**No**		**Yes**	**Missing**	**No**		**Yes**	**Missing**	**No**		**Yes**	**Missing**
	Gastroesophageal reflux ^A^	42 (36 8)		4 (3.5)	68 (59.6)	40 (43.0)		7 (7.5)	46 (49.5)	0		0	13 (100.0)
	Respiratory morbidity ^B^	105 (92.1)		9 (7.9)	0	60 (64.5)		33 (33.5)	0	12 (92.3)		1 (7.7)	0
	Daily medication for physical condition	87 (76.3)		27 (23.7)	0	70 (75.3)		23 (24.7)	0	11 (84.6)		2 (15.4)	0
	Daily medication for psychological condition	111 (97.4)		3 (2.6)	0	92 (98.9)		1 (1.1)	0	12 (92.3)		1 (7.7)	0
	Scoliosis	108 (94.5)		6 (5.3)	0	88 (94.6)		5 (5.4)	0	13 (100.0)		0	0
	Tube feeding	112 (98.2)		2 (1.8)	0	89 (95.7)		4 (4.3)	0	13 (100.0)		0	0
	Home oxygen	114 (100.0)		0	0	92 (98.9)		1 (1.1)	0	13 (100.0)		0	0

^A^ These assessments were introduced in the follow-up program at a later stage, which led to a relatively large amount of missing data. ^B^ Defined as ≥3 lower respiratory tract infections in the past year requiring antibiotic therapy and/or hospitalization, or presence of vocal cord dysfunction or subglottic stenosis requiring regular follow-up.

**Table 3:** Results of multiple linear regression analyses for PedsQL and DUX-25 scores and subscales of children with congenital diaphragmatic hernia (CDH, n = 114) and esophageal atresia (EA, n = 93). Asterisk (*) indicates significance (*p* < 0.05). B = unstandardized coefficient.

		Independent Variables													
		Cognition				Behavior				Motor Function		Maximum Exercise Capacity		Lung Function	
		Borderline (n = 21)		Impaired (n = 4)		Borderline (n = 11)		Impaired (n = 15)		Impaired (n = 37)		Impaired (n = 52)		Impaired (n = 42)	
**Dependent variables**		B	*p*-value	B	*p*-value	B	*p*-value	B	*p*-value	B	*p*-value	B	*p*-value	B	*p*-value
**CDH**	**PedsQL**														
	Physical functioning	−16.57	0.042 *	−24.15	0.14	−7.02	0.51	−5.03	0.59	0.35	0.96	−10.15	0.15	−4.65	0.46
	Emotional functioning	−17.36	0.031 *	−23.17	0.15	−7.20	0.49	−5.79	0.53	2.11	0.76	−1.22	0.86	−5.09	0.41
	Social functioning	−19.91	0.017 *	−31.21	0.059	−6.89	0.51	−8.89	0.35	−2.53	0.72	−5.97	0.41	−5.75	0.38
	School functioning	−14.63	0.054	−31.79	0.041 *	−13.71	0.18	−8.39	0.33	−2.33	0.72	−0.14	0.98	−4.16	0.48
	Psychosocial health	−17.38	0.021 *	−28.60	0.056	−8.17	0.40	−7.84	0.36	−0.41	0.95	−3.72	0.57	−4.82	0.41
	Total score	−17.44	0.022 *	−26.76	0.079	−8.87	0.36	−6.63	0.44	−0.26	0.97	−5.06	0.43	−4.86	0.41
	**DUX-25**														
	Physical functioning	3.20	0.71	−13.24	0.45	9.56	0.36	−1.81	0.86	10.81	0.13	−9.83	0.18	−1.05	0.87
	Home functioning	−1.30	0.88	−20.49	0.28	17.58	0.096	1.14	0.91	12.41	0.086	−9.87	0.18	0.89	0.90
	Emotional functioning	−0.20	0.98	−20.42	0.19	7.39	0.44	−4.19	0.64	13.60	0.037 *	−7.04	0.29	1.24	0.84
	Social functioning	−6.50	0.44	−21.41	0.20	12.84	0.21	−0.49	0.96	11.51	0.097	−8.23	0.24	−0.08	0.99
	Close social functioning	−5.86	0.51	−32.69	0.065	14.10	0.19	−2.04	0.84	13.30	0.067	−11.42	0.12	1.11	0.87
	Far social functioning	−6.47	0.44	−14.27	0.41	12.54	0.21	2.68	0.78	10.27	0.14	−5.20	0.46	−0.63	0.92
	Total score	−0.82	0.92	−18.72	0.26	10.58	0.30	−2.01	0.83	12.00	0.071	−8.95	0.19	0.41	0.95
		Borderline (n = 9)		Impaired (n = 1)		Borderline (n = 15)		Impaired (n = 5)		Impaired (n = 25)		Impaired (n = 43)		Impaired (n = 37)	
		B	*p*-value	B	*p*-value	B	*p*-value	B	*p*-value	B	*p*-value	B	*p*-value	B	*p*-value
**EA**	**PedsQL**														
	Physical functioning	−2.76	0.78	−62.34	0.032 *	−3.56	0.64	−23.65	0.092	−10.24	0.095	−3.10	0.63	−7.60	0.19
	Emotional functioning	−3.62	0.72	−56.38	0.089	4.67	0.54	−20.03	0.13	−1.43	0.82	4.83	0.45	−7.66	0.18
	Social functioning	1.82	0.87	−63.43	0.043 *	−5.67	0.49	−27.30	0.090	−6.46	0.34	−0.60	0.93	−5.02	0.42
	School functioning	−4.18	0.68	−52.31	0.055	−4.43	0.54	−30.54	0.025 *	−7.03	0.22	1.07	0.87	−7.62	0.16
	Psychosocial health	−1.44	0.89	−56.46	0.063	−0.94	0.90	−24.97	0.058	−4.29	0.47	1.14	0.85	−7.05	0.20
	Total score	−2.82	0.77	−58.90	0.044 *	−1.50	0.83	−29.16	0.029 *	−6.32	0.28	1.20	0.84	−6.50	0.23
	**DUX-25**														
	Physical functioning	−11.45	0.41	24.15	0.45	−4.88	0.67	−13.59	0.48	9.10	0.28	2.68	0.73	4.78	0.54
	Home functioning	−16.20	0.23	14.38	0.67	−7.52	0.52	−2.60	0.89	12.84	0.13	3.42	0.66	6.68	0.39
	Emotional functioning	−6.34	0.62	19.94	0.49	−7.69	0.43	−0.89	0.96	12.93	0.090	2.01	0.78	5.81	0.40
	Social functioning	−7.07	0.58	8.42	0.79	−4.57	0.67	−6.74	0.69	12.60	0.11	4.97	0.50	5.87	0.42
	Close social functioning	−6.19	0.66	7.97	0.80	−9.15	0.41	−5.19	0.79	12.85	0.12	3.40	0.60	10.03	0.19
	Far social functioning	−5.60	0.67	6.86	0.83	−1.77	0.87	−15.27	0.39	11.74	0.13	7.17	0.33	4.10	0.56
	Total score	−10.15	0.42	17.59	0.57	−6.44	0.55	−3.91	0.81	11.69	0.13	2.92	0.69	5.92	0.41

## Data Availability

Data presented in this study are available on request from the corresponding author.

## Supplementary Materials

The following are available online at https://www.mdpi.com/article/10.3390/children8070587/s1, S1: Detailed description of instruments used to measure clinical outcomes, S2: Results of multivariable linear regression analysis adjusted for sex and maternal educational level.
