# Supplementary material for: Patient-Reported Outcome Measures and Clinical Outcomes in Children with Foregut Anomalies
Source: Children (Basel). 2021 Jul 10;8(7):587. doi: 10.3390/children8070587 (PMC8307606; doi:10.3390/children8070587)
Supplement: Supplementary file 1 [file children-08-00587-s001.zip › children-1289354-supplementary.pdf]

Supplementary material of the manuscript

Patient-reported outcome measures and clinical outcomes in children with foregut anomalies

Authors and affiliations:

Isabel I. Sreeram, MD[1#], Chantal A. ten Kate, MD[1#], Joost van Rosmalen, PhD[2,3], Johannes M. Schnater, PhD[1], Saskia J. Gischler, PhD[1], René M.H. Wijnen, PhD[1], Hanneke IJsselstijn, PhD[1], André B. Rietman, PhD[1,4]

[#] Shared first author, both authors contributed equally.

[1] Department of Pediatric Surgery and Intensive Care, Erasmus MC Sophia Children's Hospital, Rotterdam, the Netherlands

[2] Department of Biostatistics, Erasmus University Medical Centre, the Netherlands

[3] Department of Epidemiology, Erasmus University Medical Centre, the Netherlands

[4] Department of Child and Adolescent Psychiatry/Psychology, Erasmus MC Sophia Children's Hospital, Rotterdam, the Netherlands

Corresponding author:

André Rietman, PhD, a.rietman@erasmusmc.nl

Contents:

S1. Detailed description of instruments used to measure clinical outcomes

S2. Results for multivariable linear regression analysis adjusted for sex and maternal educational level

S1. Detailed description of instruments used to measure clinical outcomes

Health status

Pediatric Quality of Life Inventory (PedsQL)[1]

The PedsQL questionnaire is an instrument for assessing health status in children, adolescents, and adults, of which different age-appropriate versions are available. It consists of 23 items, divided into four subscales: physical functioning (eight items), emotional functioning (five items), social functioning (five items), and school functioning (five items). A fifth subscale, psychosocial health, is the sum of the emotional, social, and school functioning scales. The total score is calculated from all 23 items together. Answers range from 0 (never a problem) to 4 (almost always a problem) and are converted to a score between 0 and 100, with higher scores representing better health status.

In 2009, the PedsQL was validated in Dutch children aged 5-18 years[2], and sex-specific norm values have recently been updated [manuscript submitted]. See Supplementary Table 1.

Quality of life

Dutch-Child-AZL-TNO-Quality-of-Life (DUX-25) [3]

The DUX-25 questionnaire is an instrument used to measure quality of life in children aged five years and older. It is comprised of 25 items within four domains: physical functioning (six items), emotional functioning (seven items), social functioning (seven items), and home functioning (five items). Social functioning can be divided into close and far social functioning. Close social functioning involves interaction with peers (e.g., friends or classmates). Far social functioning involves interaction with adults or teachers and the child's feelings towards other children. The total score is calculated from all 25 items together. Items are answered using a visual 5-point Likert scale, which are then converted to a score between 0-100. Higher scores indicate a better quality of life.

The DUX-25 has been modified from the original TNA-AZL Children's quality of life questionnaire (TACQOL), which contained 54 items for the self-report and 63 items for the parent-report[4]. Recently, the DUX-25 has been validated in Dutch children aged 8-17 years, with updated sex-specific norm values [manuscript submitted]. See Supplementary Table 2.

Cognition

Revised Amsterdam Intelligence Test (RAKIT) [5]

The RAKIT is an instrument for measuring intelligence (IQ) in children aged 4-12 years. It consists of 12 subtests, which provide – besides a total IQ score – scores for perceptual reasoning, verbal learning, spatial orientation, and verbal fluency.

The RAKIT was developed in the Dutch language and has been validated in Dutch children. In our hospital, the RAKIT was used until the end of 2009, after which it was replaced by the WISC. For this study, only total IQ scores were used. IQ >85 was considered normal, IQ 70-84 was considered borderline, and IQ <70 was considered impaired.

Wechsler Intelligence Scale for Children (WISC)[6]

The WISC is an instrument for measuring IQ in children aged 6-17 years. The Dutch WISC-III-NL consists of 13 subtests, which measure verbal, performance, and total IQ. The Dutch WISC-V-NL consists of 14 subtests, including eight tests from the 3rd version.

Both editions have been validated in Dutch children, with a mean (SD) of 100 (15)[6, 7]. In our hospital, the WISC-III-NL was used until the end of 2019, after which it was replaced by the WISC-V-NL. For this study, only total IQ scores were used. IQ >85 was considered normal, IQ 70-84 was considered borderline, and IQ <70 was considered impaired.

Behavior

Strengths and Difficulties Questionnaire (SDQ) [8]

The SDQ is a brief emotional and behavioral screening tool for children aged between two and 17 years. A proxy-report for parents and teachers is available for children aged two years and older. The SDQ consists of 25 items divided between five domains: emotional symptoms (five items), conduct problems (five items), hyperactivity and inattention (five items), peer relationships (five items), and prosocial behavior (five items). A total score is calculated from all 25 items together.

For this study, we used the Dutch version of the parental proxy-reports[9]. The SDQ has been part of our follow-up program since 2011. We determined our cut-off values based on sex-dependent mean (SD) total scores. Up to +1SD was deemed normal, +1SD to +2SD was deemed borderline, and a score of >+2SD was deemed impaired.

Child Behavior Checklist (CBCL)[10, 11]

The CBCL is an instrument to rate behavioral and emotional problems in children, and is completed by parents or caretakers. Two versions are available: one for children aged 2-3 years and one for children aged 4-16 years. It consists of 20 items regarding activities, social interaction, and school functioning, 118 items regarding emotions and behavior, and two open questions on other behavioral problems. Answers are scored on a three-point scale and include not at all, sometimes, and often. The complete checklist results in a syntax-calculated total score.

For this study, we used parental proxy-reports for children aged 4-16 years. The CBCL was used in our follow-up program until the end of 2010. Based on Dutch norm values, behavior was scored as normal, borderline or impaired[12].

Daily executive functioning

Behavior Rating Inventory of Executive Function (BRIEF)[13]

The BRIEF is a rating scale for assessing the everyday behavioral manifestations of the child's executive control functions. Three versions are available: a parent-reported version for children aged 5-17 years, a teacher-reported version for children aged 5-11 years, and a self-reported version for children aged 11-17 years. The proxy-reports consist of 75 items, whereas the self-report consists of 68 items. These items are divided into eight scales: inhibition, flexibility, regulation of emotions, initiative, working memory, planning, organization of materials, and monitoring. The scales are summarized into two indices (behavior and metacognition) and a total score. The BRIEF has been implemented in our follow-up programme from 2015 onwards.

For this study, we evaluated the total scores of validated Dutch parental proxy-reports[14]. A total score of <60 is considered normal, a total score of 60-65 is considered borderline and a total score of >65 is considered impaired.

Motor function

Movement Assessment Battery for Children (MABC)[15]

The MABC is a motor function test and is used to evaluate a child's motor function. The MABC-I is used for children aged 4-12 years and the second version, the MABC-II, can be used for children aged 3-16 years. The checklist, that consists of 48 items for the MABC-I and 30 items for the MABC-II, covers activities in daily life and can be filled in by parents or teachers. The motor function test consists of eight items divided into three domains: three manual dexterity tasks, two ball-skill tasks, and three balance tasks. Each item is scored from 0 to 5 (good to very poor). This results in domain-specific scores and a total impairment score, which can be interpreted as a percentile using age-specific normative data tables.

In our follow-up program, the MABC-I was used until 2012, after which it was replaced by the MABC-II. Both editions contain similar content, are assumed to be comparable, and have been validated in Dutch children[16, 17]. We evaluated the percentiles of the total impairment scores. A percentile of ≥16 was considered normal and a percentile of ≤15 was considered impaired.

Maximum exercise capacity

Bruce protocol[18]

The Bruce protocol is a treadmill test for evaluating exercise capacity. During the test, the incline and speed are adjusted every three minutes according to the standardized Bruce protocol and the child is encouraged to perform until exhaustion. Before and during the test, both heart rate and transcutaneous oxygen saturation are monitored. Maximal performance or exhaustion is defined as a heart rate of ≥185 beats per minute or loss of coordination.

In our study, the maximal endurance time in minutes was compared to recently defined age-specific reference values for Dutch children, leading to an SD score[19]. SD scores of ≥-1 was considered normal and <-1 was considered impaired.

Lung function

Lung function was tested according to criteria set by the European Respiratory Society[20]. Results were categorized as normal or impaired (standard deviation (SD) <-1.64 or >1.64) based on the forced expiratory volume (FEV1) before bronchodilation, in accordance with the Global Lung Initiative 2012 [21].

Maternal educational level (MEL)

MEL was used as a proxy for socioeconomic status and categorized according to the International Standard Classification of Education[22].

		Median (IQR)	
**Self-reports**		*Male (n=93)*	*Female (n=146)*
	Physical functioning	93.75 (90.63-100.00)	90.63 (84.38-96.88)
	Emotional functioning	80.00-65.00-85.00)	75.00 (60.00-85.00)
	Social functioning	90.00 (75.00-100.00)	90.00 (80.00-100.00)
	School functioning	80.00 (75.00-90.00)	85.00 (75.00-95.00)
	Psychosocial health	81.67 (75.00-88.33)	83.33 (76.67-90.00)
	Total score	85.87 (80.44-91.30)	85.87 (79.35-91.30)
**Proxy-reports**		*Male (n=121)*	*Female (n=179)*
	Physical functioning	93.75 (84.38-100.00)	90.63 (84.38-96.88)
	Emotional functioning	75.00 (65.00-82.50)	75.00 (60.00-90.00)
	Social functioning	90.00 (75.00-100.00)	90.00 (75.00-100.00)
	School functioning	75.00 (65.00-90.00)	85.00 (75.00-95.00)
	Psychosocial health	78.33 (71.67-86.67)	81.67 (73.33-90.00)
	Total score	83.70 (77.17-90.22)	84.78 (77.17-91.30)

Supplementary Table 1. Sex-specific Dutch norm values for PedsQL scales for 8-to-12-year-old healthy children. IQR = interquartile range.

		Median (IQR)	
**Self-reports**		*Male (n=92)*	*Female (n=145)*
	Physical functioning	89.58 (79.17-95.83)	87.50 (75.00-95.83)
	Home functioning	95.00 (90.00-100.00)	95.00 (90.00-100.00)
	Emotional functioning	78.57 (67.86-89.29)	82.14 (67.86-89.29)
	Social functioning	82.14 (71.43-89.29)	82.14 (75.00-89.29)
	Social close functioning	91.67 (83.33-100.00)	91.67 (83.33-100.00)
	Social far functioning	75.00 (62.50-87.50)	75.00 (68.75-87.50)
	Total functioning	85.50 (76.00-92.00)	85.00 (77.50-90.00)
**Proxy-reports**		*Male (n=121)*	*Female (n=179)*
	Physical functioning	91.67 (75.00-100.00)	91.67 (75.00-96.88)
	Home functioning	95.00 (80.00-100.00)	95.00 (80.00-100.00)
	Emotional functioning	82.14 (71.43-92.86)	85.71 (71.43-92.86)
	Social functioning	85.71 (71.43-92.86)	85.71 (75.00-96.43)
	Social close functioning	91.67 (75.00-100.00)	91.67 (75.00-100.00)
	Social far functioning	81.25 (62.50-93.75)	82.25 (68.75-93.75)
	Total functioning	86.00 (74.00-94.50)	87.00 (75.00-94.00)

Supplementary Table 2. Sex-specific Dutch norm values for DUX-25 scales for 8-to-12-year-old healthy children. IQR = interquartile range.

S2. Results of multivariable linear regression analysis adjusted for sex and maternal educational level

		Independent variables																			
		**Sex**		**Maternal educational level**				**Cognitive functioning**				**Behavior**				**Motor function**		**Exercise capacity**		**Lung function**	
		Male (n=70)		Middle (n=43)		Low (n=8)		Borderline (n=21)		Impaired (n=4)		Borderline (n=11)		Impaired (n=15)		Impaired (n=37)		Impaired (n=52)		Impaired (n=42)	
**Dependent variables**		B	*p*-value	B	*p*-value	B	*p*-value	B	*p*-value	B	*p*-value	B	*p*-value	B	*p*-value	B	*p*-value	B	*p*-value	B	*p*-value
**CDH**	**PedsQL**																				
	Physical functioning	0.57	0.94	-2.62	0.74	-19.90	0.16	-16.14	0.056	-22.39	0.19	-4.11	0.70	-4.72	0.62	3.28	0.67	-9.83	0.11	-1.05	0.50
	Emotional functioning	-5.37	0.46	0.96	0.90	-3.13	0.83	-17.78	0.033 *	-26.13	0.12	-6.26	0.54	-6.25	0.53	2.43	0.75	-9.87	0.97	0.89	0.37
	Social functioning	-3.08	0.68	-2.22	0.78	-22.03	0.11	-19.92	0.021 *	-29.10	0.091	-5.21	0.62	-10.65	0.28	1.07	0.89	-7.04	0.35	1.24	0.37
	School functioning	-6.33	0.36	0.25	0.97	-17.69	0.20	-15.08	0.055	-32.54	0.049 *	-11.50	0.24	-9.63	0.29	0.14	0.99	-8.23	0.87	-0.08	0.42
	Psychosocial health	-5.19	0.46	-0.05	1.00	-14.06	0.34	-17.69	0.023 *	-30.56	0.061	-8.57	0.38	-8.48	0.35	1.03	0.89	-11.42	0.88	1.11	0.34
	Total score	-3.01	0.66	-1.06	0.88	-15.57	0.23	-17.37	0.028 *	-25.91	0.19	-6.89	0.49	-7.62	0.40	2.08	0.77	-5.20	0.51	-0.63	0.39
	**DUX-25**																				
	Physical functioning	11.28	0.14	8.23	0.32	1.28	0.93	0.03	1.00	-12.19	0.51	9.96	0.35	3.70	0.72	7.76	0.31	-11.39	0.13	0.32	0.96
	Home functioning	9.01	0.25	8.19	0.32	-6.06	0.70	-3.58	0.70	-17.39	0.36	18.00	0.091	5.20	0.62	10.46	0.18	-11.45	0.14	2.17	0.75
	Emotional functioning	4.99	0.48	8.15	0.28	-10.81	0.41	-2.81	0.73	-19.01	0.25	8.27	0.40	-1.39	0.89	12.49	0.08	-8.35	0.24	1.78	0.78
	Social functioning	5.84	0.43	8.33	0.29	-9.18	0.51	-9.33	0.28	-19.86	0.27	13.56	0.18	1.82	0.86	10.25	0.18	-9.22	0.21	0.85	0.90
	Close social functioning	5.29	0.50	4.65	0.54	-9.30	0.58	-7.83	0.39	-31.36	0.10	14.64	0.17	-1.26	0.91	12.59	0.12	-12.72	0.10	1.69	0.81
	Far social functioning	6.95	0.35	12.49	0.11	-9.01	0.52	-10.67	0.22	-13.36	0.46	15.12	0.15	15.12	0.51	7.60	0.32	-6.74	0.36	0.37	0.96
	Total score	7.86	0.28	9.70	0.20	-7.01	0.62	-4.83	1.00	-16.90	0.51	13.50	0.15	3.70	0.72	9.77	0.18	-10.10	0.15	1.32	0.84
		Male (n=57)		Middle (n=37)		Low (n=15)		Borderline (n=9)		Impaired (n=1)		Borderline (n=15)		Impaired (n=5)		Impaired (n=25)		Impaired (n=43)		Impaired (n=37)	
		B	*p*-value	B	*p*-value	B	*p*-value	B	*p*-value	*p*-value	B	B	*p*-value	B	*p*-value	B	*p*-value	B	*p*-value	B	*p*-value
**EA**	**PedsQL**																				
	Physical functioning	0.80	0.89	-7.32	0.24	-13.07	0.14	-1.57	0.88	-52.25	0.068	-1.28	0.87	-12.29	0.29	-7.30	0.25	-4.16	0.50	-9.19	0.11
	Emotional functioning	-1.40	0.81	-9.46	0.12	-7.26	0.43	-3.78	0.74	-53.81	0.13	6.47	0.42	-15.19	0.18	0.07	0.99	4.82	0.44	-7.72	0.18
	Social functioning	3.04	0.63	-12.11	0.069	-12.23	0.20	5.06	0.67	-62.10	0.053	-2.19	0.80	-16.62	0.15	-3.17	0.64	-1.84	0.79	-5.78	0.35
	School functioning	-1.78	0.75	-8.46	0.15	-10.04	0.24	-0.97	0.93	-55.30	0.056	-2.02	0.78	-19.62	0.048 *	-5.02	0.39	-0.22	0.97	-8.96	0.092
	Psychosocial health	0.05	0.99	-10.34	0.073	-10.42	0.22	0.89	0.94	-54.29	0.080	0.56	0.94	-16.02	0.16	-2.79	0.64	1.07	0.86	-7.41	0.17
	Total score	0.28	0.96	-9.21	0.12	-10.36	0.24	-0.52	0.96	-59.83	0.056	0.71	0.92	-15.15	0.19	-4.34	0.46	-0.51	0.93	-7.84	0.15
	**DUX-25**																				
	Physical functioning	1.00	0.90	3.48	0.68	-5.55	0.65	-10.56	0.48	27.00	0.43	-5.61	0.62	-15.70	0.37	10.00	0.24	3.74	0.65	3.10	0.70
	Home functioning	-4.52	0.58	0.64	0.94	-5.59	0.64	-16.01	0.30	17.51	0.61	-5.87	0.60	-3.15	0.88	13.46	0.12	4.50	0.59	5.85	0.47
	Emotional functioning	-1.12	0.88	3.72	0.64	-4.43	0.69	-6.66	0.65	25.60	0.40	-9.75	0.34	0.12	1.00	13.39	0.085	2.58	0.73	4.29	0.55
	Social functioning	-0.49	0.95	2.13	0.79	0.02	1.00	-2.76	0.86	8.17	0.79	-6.20	0.55	-23.50	0.18	11.97	0.13	8.08	0.31	6.67	0.37
	Close social functioning	-3.88	0.63	1.37	0.87	-3.46	0.77	-7.48	0.61	9.86	0.77	-9.72	0.42	-8.14	0.65	13.34	0.12	5.05	0.53	9.38	0.24
	Far social functioning	-0.16	0.98	0.23	0.98	2.85	0.80	-6.70	0.65	2.47	0.94	-3.07	0.77	-22.05	0.19	11.17	0.16	9.81	0.22	4.18	0.56
	Total score	-0.95	0.90	1.96	0.81	-4.75	0.67	-8.14	0.57	22.34	0.48	-6.35	0.54	-6.99	0.68	12.20	0.13	4.04	0.59	4.83	0.52

Supplementary Table 3. Results of multivariable regression analyses of PedsQL and DUX-25 scores and subscales of children with congenital diaphragmatic hernia (CDH, n=114) and esophageal atresia (EA, n=93). All models included sex, maternal education level, cognition, behavior, motor function, maximum exercise capacity and lung function as independent variables. *Asterisk indicates significance (p<0.05). B = unstandardized coefficient

References

1. Varni JW, Seid M, Kurtin PS. PedsQL 4.0: reliability and validity of the Pediatric Quality of Life Inventory version 4.0 generic core scales in healthy and patient populations. Med Care. 2001;39(8):800-12.
2. Engelen V, Haentjens MM, Detmar SB, Koopman HM, Grootenhuis MA. Health related quality of life of Dutch children: psychometric properties of the PedsQL in the Netherlands. BMC Pediatr. 2009;9:68.
3. Koopman HM TN, Vogels AGC, Kamphuis RP, Verrips GH. The DUC-25: a short-form questionnaire for measuring health related quality of life of children with a chronic illness. Quality of Life Research. 1998;7:619.
4. Kolsteren MM, Koopman HM, Schalekamp G, Mearin ML. Health-related quality of life in children with celiac disease. J Pediatr. 2001;138(4):593-5.
5. Bleichrodt N, Drenth PJD, Zaal JM, Resing WCM. Intelligentiemeting bij Kinderen. Lisse, The Netherlands: Zwets en Zeitlinger; 1987.
6. Kort W, Schittekatte M, Bosmans M, Compaan EL, Dekker PH, Vermeir G, et al. Wechsler Intelligence Scale for Children-III, Dutch version. Amsterdam: Pearson; 2005.
7. Hendriks MPH, Ruiter S. Wechsler Intelligence Scale for Children-V, Dutch version. Amsterdam: Pearson; 2017.
8. Goodman R. The Strengths and Difficulties Questionnaire: a research note. J Child Psychol Psychiatry. 1997;38(5):581-6.
9. van Widenfelt BM, Goedhart AW, Treffers PD, Goodman R. Dutch version of the Strengths and Difficulties Questionnaire (SDQ). Eur Child Adolesc Psychiatry. 2003;12(6):281-9.
10. Achenbach TM. The Child Behavior Profile: I. Boys aged 6--11. J Consult Clin Psychol. 1978;46(3):478-88.
11. Achenbach TM, Edelbrock CS. The Child Behavior Profile: II. Boys aged 12-16 and girls aged 6-11 and 12-16. J Consult Clin Psychol. 1979;47(2):223-33.
12. Verhulst F, Koot J, Akkerhuis G, Veerman J. Praktische handleiding voor de CBCL (Child Behavior Checklist). Assen: Van Gorcum; 1990.
13. Gioia GA, Isquith PK, Retzlaff PD, Espy KA. Confirmatory factor analysis of the Behavior Rating Inventory of Executive Function (BRIEF) in a clinical sample. Child Neuropsychol. 2002;8(4):249-57.
14. Smidts D, Huizinga M. BRIEF Vragenlijst executieve functies voor 5- tot 18-jarigen. Amsterdam: Hogrefe Uitgevers BV; 2012.
15. Henderson S, Sugden D. The movement assessment battery for children: manual.: The Psychological Corporation; 1992.
16. Smits-Engelsman B, Niemeijer A. Movement Assessment Battery for Children, tweede editie (Movement ABC-2). Nederlands tijdschrift voor Kinderfysiotherapie. 2010;64:9-13.
17. Smits-Engelsman BC, Fiers MJ, Henderson SE, Henderson L. Interrater reliability of the Movement Assessment Battery for Children. Phys Ther. 2008;88(2):286-94.
18. Bruce RA, Kusumi F, Hosmer D. Maximal oxygen intake and nomographic assessment of functional aerobic impairment in cardiovascular disease. Am Heart J. 1973;85(4):546-62.
19. van der Cammen-van Zijp MH, van den Berg-Emons RJ, Willemsen SP, Stam HJ, Tibboel D, IJsselstijn H. Exercise capacity in Dutch children: new reference values for the Bruce treadmill protocol. Scand J Med Sci Sports. 2010;20(1):e130-6.
20. Miller MR, Hankinson J, Brusasco V, Burgos F, Casaburi R, Coates A, et al. Standardisation of spirometry. Eur Respir J. 2005;26(2):319-38.
21. Quanjer PH, Stanojevic S, Cole TJ, Baur X, Hall GL, Culver BH, et al. Multi-ethnic reference values for spirometry for the 3-95-yr age range: the global lung function 2012 equations. Eur Respir J. 2012;40(6):1324-43.

22. UNESCO Institute of Statistics. International Standard Classification of Education (ISCED) 2011 [Available from: http://uis.unesco.org/en/topic/international-standard-classification-education-isced.
